# Role of inflammatory markers in the diagnosis of vascular contributions to cognitive impairment and dementia: a systematic review and meta-analysis

**DOI:** 10.1007/s11357-022-00556-w

**Published:** 2022-04-29

**Authors:** Carlo Custodero, Alessandro Ciavarella, Francesco Panza, Davide Gnocchi, Gennaro M. Lenato, Juhan Lee, Antonio Mazzocca, Carlo Sabbà, Vincenzo Solfrizzi

**Affiliations:** 1 Dipartimento Interdisciplinare di Medicina, Clinica Medica e Geriatria “Cesare Frugoni”, University of Bari Aldo Moro, Bari, Italy; 2 Fondazione IRCCS Ca’ Granda—Ospedale Maggiore Policlinico, A. Bianchi Bonomi Hemophilia and Thrombosis Center, Milan, Italy; 3 Population Health Unit—“Salus In Apulia Study”, National Institute of Gastroenterology “Saverio de Bellis”, Research Hospital, Castellana Grotte, Bari, Italy; 4 Department of Psychiatry, Yale University School of Medicine, New Haven, CT USA

**Keywords:** Vascular contributions to cognitive impairment and dementia, Alzheimer’s disease, Interleukin-6, C-reactive protein, Tumor necrosis factor-α

## Abstract

Vascular contribution to cognitive impairment and dementia (VCID) is a clinical label encompassing a wide range of cognitive disorders progressing from mild to major vascular cognitive impairment (VCI), which is also defined as vascular dementia (VaD). VaD diagnosis is mainly based on clinical and imaging findings. Earlier biomarkers are needed to identify subjects at risk to develop mild VCI and VaD. In the present meta-analysis, we comprehensively evaluated the role of inflammatory biomarkers in differential diagnosis between VaD and Alzheimer’s disease (AD), and assessed their prognostic value on predicting VaD incidence. We collected literature until January 31, 2021, assessing three inflammatory markers [interleukin(IL)-6, C-reactive protein (CRP), tumor necrosis factor (TNF)-α] from blood or cerebrospinal fluid (CSF) samples. Thirteen cross-sectional and seven prospective studies were included. Blood IL-6 levels were cross-sectionally significantly higher in people with VaD compared to AD patients (SMD: 0.40, 95% CI: 0.18 to 0.62) with low heterogeneity (*I*^2^: 41%, *p* = 0.13). Higher IL-6 levels were also associated to higher risk of incident VaD (relative risk: 1.28, 95% CI: 1.03 to 1.59, *I*^2^: 0%). IL-6 in CSF was significantly higher in people with VaD compared to healthy subjects (SMD: 0.77, 95% CI: 0.17 to 1.37, *I*^2^: 70%), and not compared to AD patients, but due to limited evidence and high inconsistency across studies, we could not draw definite conclusion. Higher blood IL-6 levels might represent a useful biomarker able to differentiate people with VaD from those with AD and might be correlated with higher risk of future VaD.

**Supplementary Information:**

The online version contains supplementary material available at 10.1007/s11357-022-00556-w.

## Introduction

Vascular contributions to cognitive impairment and dementia (VCID) are conditions arising from vascular diseases or abnormalities that result in a wide range of cognitive disorders progressing from mild to major vascular cognitive impairment (VCI), which is also defined as vascular dementia (VaD) [1, 2]. Among the different forms of dementia, VaD is considered the second most common cause after Alzheimer’s disease (AD), accounting approximately the 20% of dementia cases [2]. Its prevalence is estimated to be 0.6–2.1% in subjects aged over 65 years [3], and it increases with age, up to 4.8% in those over 85 years [4]. Cerebrovascular disease is the main etiological feature of VCID, independent of the underlying mechanism (e.g., multiple or single territorial or small infarcts, strategic infarcts) and the occurrence of stroke symptoms [5]. However, it is becoming clear that white matter damage and cognitive impairment occur also in absence of stroke symptoms, suggesting that often there is a silent and slow progression of the disease due to involvement of cerebral small vessels [6]. For example, VCID can be detected in subjects suffering from arrhythmias (e.g., atrial fibrillation) or other vascular risk factors (e.g., tobacco use, hypertension, obesity, hyperlipidemia, diabetes, hyper-coagulation), but without stroke history. In addition, vascular manifestations in older adults often fluctuate over time, resulting in diagnostic delay and ineffective treatments [7].

The main cerebrovascular signs of VCID are brain atrophy, white matter hyperintensities (WMH) lesions, infarctions, and hemorrhages. Indeed, imaging techniques (e.g., magnetic resonance imaging or computed tomography) represent an essential step in the diagnosis and evaluation of disease progression. However, evidence at neuroimaging is already a sign of advanced stage of disease and non-reversible brain damage. As for the definition of AD with introduction of amyloid, tau, neurodegeneration (AT[N]) system [8], also for VCID, there is a need to change the paradigm traditionally based on clinical history and signs and symptoms of the disease, toward a biological framework founded on early biomarkers able to predict development of VCID. This approach could allow the recognition of subjects at risk in a preclinical phase and timely the implementation of potential preventive strategies.

To date, no specific circulating biomarker is available in the diagnosis of VCID [9]. Over the last several years, there has been growing interest in addressing the relationship between inflammation, cardiovascular diseases, and cognitive dysfunction [10]. It is becoming clear that inflammation may have a role in the pathogenesis of dementia. As already reviewed, promising perspectives came from inflammatory biomarkers in predicting risk of overall dementia [11]. Previous meta-analyses showed that increased circulating interleukin(IL)-6 and C-reactive protein (CRP) levels were associated to higher risk of dementia from all causes, but not to AD [12, 13]. These studies did not test the role of inflammatory biomarkers in differential diagnosis between VaD and AD, and did not include inflammatory markers from cerebrospinal fluid (CSF). We hypothesized that inflammatory biomarkers may be increased to a greater extent in VCID compared to AD; thus, the goal of this review was to examine the diagnostic and predictive power of selected inflammatory biomarkers for VCID.

## Methods

### Search strategy and study selection

The present systematic literature review and meta-analysis followed the requirements of the PRISMA statement [14]. An a priori protocol was established and registered on PROSPERO, an international prospective register of systematic reviews (http://www.crd.york.ac.uk/PROSPERO; registration number: CRD42021268548).

Two study authors (C.C. and A.C.) independently conducted a systematic search of the databases MEDLINE, PubMed, Scopus, Web of Science, and Google Scholar until January 31, 2021. Search terms included combinations of the following keywords: (“C-reactive protein” OR “interleukin-6” OR “tumor necrosis factor-α” OR “inflammation” OR “inflammatory marker”) AND ((“blood” AND “vessels”) OR “blood vessels” OR “vascular”)) AND (“cognitive impairment” OR “dementia”). To be included in the present meta-analysis, studies had to be observational with either cross-sectional or longitudinal design. Studies were required to meet the following inclusion criteria: (1) conducted in humans; (2) assessed at least one specific inflammatory marker in serum, plasma or CSF; (3) including subjects with some form of VCID; (4) written in the English language. In addition, studies with the following characteristics were excluded: (1) interventional studies; (2) with not clear differentiation among dementia subtypes; (3) lack of comparison group; (4) prospective studies including subjects with dementia at baseline; (5) autoptic studies. Articles were initially screened based on title and abstract by two study authors (C.C., and A.C.), with the full text sought if the abstract did not provide sufficient information. Reference lists of the articles were reviewed to identify additional relevant articles. Disagreement was resolved by discussion or in consultation with a senior author (V.S.). We contacted the authors of primary studies to obtain any missing information.

### Data extraction

The following details were extracted from each study: first author’s name, publication year and country, sample size, details of study population (mean age, health status), study duration, study design, assessed inflammatory markers, definition of dementia diagnosis, potential confounders that were considered in the analysis and main results. For prospective studies, we extracted the reported effect estimates (relative risk (RR) or hazard ratio (HR)) and the corresponding 95% confidence interval (CI) derived from the most fully adjusted model for potential confounders if studies reported several multivariable-adjusted RRs.

### Risk of bias assessment

The quality of the studies was assessed using appropriate tool for observational studies: the Newcastle–Ottawa Quality Assessment Scale (NOS) [15]. Two study authors (A.C. and D.G.) assigned a rating, using stars, based on three domains: selection of study population (0–4 stars), comparability of study groups (0–2 stars), ascertainment of outcome (for cohort studies) or exposure (for case–control studies) (0–3 stars). The final NOS score for each study ranges from 0 stars (lowest quality) to 9 stars (highest quality), with studies scoring 0–3 stars judged as low quality, those between 4 and 6 as medium quality, and those between 7 and 9 considered to be of high quality. Discrepancies in the evaluation were solved by discussion. The reliability of assessment was ensured by revision and consultation with a senior author (V.S.).

### Statistical analysis

For studies with cross-sectional design, each study’s effect size, or standardized mean difference (SMD), was calculated by comparing mean and standard deviation of inflammatory biomarkers, between VaD and control or AD groups [16]. If the data were reported as median and interquartile range, the correspondent mean and standard deviation were estimated using the method developed by Wan and colleagues [17]. In accordance with convention, effect sizes were classified as small (0.2), moderate (0.5), and large (0.8) [18]. For studies with longitudinal design, study-specific risk estimates were extracted from each article, and log risk estimates were weighted by the inverse of their variances to obtain a pooled risk estimate. The primary analyses combined ln RR associated with one-unit change in inflammatory markers. Studies were combined using the DerSimonian and Laird random-effects model, which considers both within- and between-study variations [16]. Heterogeneity across studies was estimated by *I*^2^ statistic. It measures percentage of variation that is caused by heterogeneity between studies, and is larger when heterogeneity increases [16]. Sensitivity analyses were performed to investigate the influence of each individual study on the overall meta-analysis summary estimate and the validity of the effect size. Further sensitivity analysis was performed to determine the robustness of findings by excluding studies with poor-quality assessment (NOS score < 7). Funnel plots and Egger’s tests were utilized to detect bias in meta-analyses [16]. Statistical analyses were performed in RevMan 5.4 (The Cochrane Collaboration, Oxford, England) and STATA 14.0 software (StataCorp LP, College Station, TX, USA). Each *p* value is based on two-sided alternative hypothesis, and a level of 0.05 or below was considered statistically significant.

## Results

### Search results and study selection

**Fig. 1:**
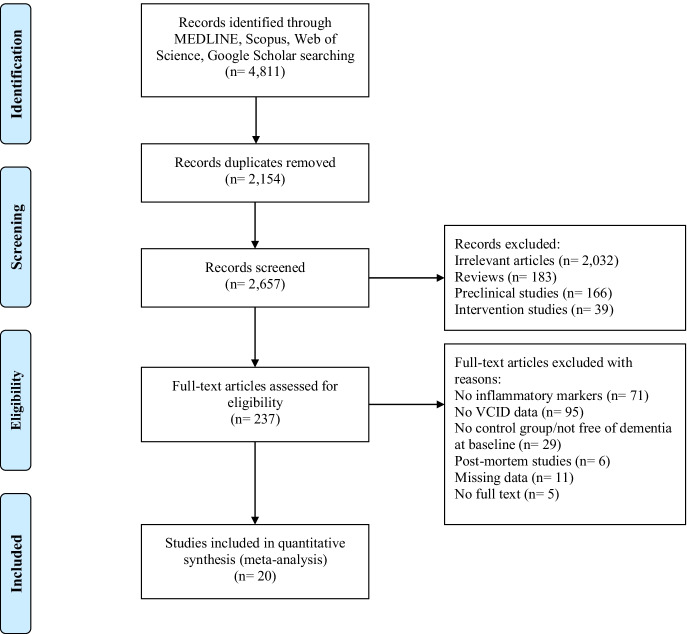
PRISMA flowchart of identification and selection of eligible studies

Details about the study selection process are shown in Fig. 1. A total of 2,657 articles were identified and screened. Two thousand four hundred twenty papers were excluded on the basis of titles and abstracts and full text of 237 papers were reviewed. Other 217 studies were excluded for absence of investigated inflammatory markers, lack of information about any form of VCID, absence of comparison group, inclusion of subjects with dementia in prospective cohort studies, missing data, or not availability of full text. Overall, 20 studies were eligible for meta-analysis, 13 case–control studies [19–31] (Table 1), and seven cohort studies [32–38] (Table 2). Seventeen articles analyzed inflammatory biomarkers on sera or plasma [19–22, 25–27, 29–38], two on CSF [23, 24], and one on both blood and CSF [28].

**Table 1:** Characteristics of cross-sectional studies investigating relationship between inflammatory markers and vascular dementia

Study (country)	Population	Sample size	Mean age	Inflammatory biomarkers	Covariates	Diagnostic criteria	Main results
Zhang, 2017 (China)	Subjects with VCI with no dementia, VaD and cognitively healthy (CON)	VaD: 30 Mild VCI: 30 CON: 30	65.7 y	CRP, IL-6, TNF-α [serum]	-	VaD: MoCA, CDR, ADL and HIS MRI scan	Higher CRP, IL-6 and TNF-α in VaD vs mild VCI or CON (*p* < 0.05)
Dukic, 2016 (Croatia)	Patients older than 60 years with AD, VaD, MCI or cognitively healthy (CON)	VaD: 67 MCI: 48 AD: 70 CON: 50	73.0 y	CRP, IL-6 [serum]	-	VaD: NINDS-AIREN criteria AD: NIA-AA 2011 criteria MCI: Petersen's criteria CT scan	Higher IL-6 in VaD vs AD, MCI or CON (*p* = 0.014) Lower CRP in AD
Uslu, 2012 (Turkey)	Newly diagnosed, randomly chosen AD, VaD patients and or cognitively healthy (CON)	VaD: 16 AD: 28 CON: 23	68.4 y	IL-6, TNF-α [serum]	-	AD: NINCDS-ADRDA criteria VaD: NINDS- AIREN criteria	IL-6 and TNF-α levels did not differ significantly across AD, VaD and CON
Li, 2010 (Germany)	Community dwelling subjects with AD, VaD, cognitive impairment, or cognitively healthy (CON)	VaD: 16 MCI: 17 AD: 28 CON: 22	78.5 y	hs-CRP [serum]	-	AD: NINCDS-ADRDA criteria VaD: NINDS- AIREN criteria MCI: Petersen's criteria	Trend toward higher hs-CRP in all disease groups (AD, VaD, MCI) vs CON
Jia, 2005 (China)	Hospitalized patients with AD, VaD, or cognitively healthy cases with peripheral nerve diseases (CON)	VaD: 38 AD: 39 CON: 35	64.2 y	IL-1α, IL-1β, IL-2, IL-6, TNF-α, T-tau, P-tau, Aβ1–42 [CSF]	-	AD: NINCDS-ADRDA criteria VaD: NINDS- AIREN criteria	Higher CSF IL-6 in AD and VaD vs CON (*p* < 0.01), no significant difference between AD and VaD Higher CSF TNF-α in AD vs CON (*p* < 0.01), and lower vs VaD (*p* < 0.01)
Wada-Isoe, 2004 (Japan)	Patients with AD, VaD, CVND, and other neurological disorders without cognitive impairment (CON)	VaD: 11 AD: 26 CVND: 11 CON: 21	69.0 y	IL-6 [CSF]	-	AD: DSM-III-R, NINCDS-ADRDA criteria and HIS ≤ 4 VaD: DSM-III-R, ADDTC criteria and HIS ≥ 7 CT scan or MRI and SPECT	Higher CSF IL-6 in VaD vs AD (*p* < 0.01) or CON (*p* < 0.01)
Paganelli, 2002 (Italy)	Community dwelling subjects with AD, VaD or mixed dementia	VaD: 18 AD: 36 (25 mild AD, 11 severe AD) MIX: 16	76.7 y	IL-1β, TNFα [serum]	-	AD: NINCDS-ADRDA criteria VaD: NINDS- AIREN criteria CT or MRI scan	Lower TNF-α in mild-moderate AD vs severe AD (*p* < 0.001), VaD (*p* < 0.001) and MIX (*p* < 0.001) Lower TNF-α/IL-1β ratio in mild-moderate AD vs VaD and MIX
De Luigi, 2001 (Switzerland)	Patients with different types of dementia, cognitive impairment, or cognitively healthy (CON)	VaD: 7 AD: 44 MIX: 10 Uncertain dementia: 18 MCI: 12 CON: 42	Not reported	TNF-α, IL-1β, sTNF-RI, IL-1Ra, IL-10 [plasma]	-	VaD: modified HIS > 4 AD: NINCDS-ADRDA criteria	Higher TNF-α, sTNF-RI in VaD vs CON (*p* < 0.001) After LPS stimulus reduced production of TNF-α in all dementia groups and of IL-10 in VaD
Zuliani, 2007 (Italy)	Patients with AD, VaD, CDND, and cognitively healthy (CON)	VaD: 80 AD: 60 CVND: 40 CON: 42	76.4 y	IL-6, TNF-α, IL-1β, IL-10 [serum]	Age, gender, coronary heart disease, diabetes, hypertension, smoking, and alcohol consumption	AD: NINCDS-ADRDA criteria VaD: NINDS- AIREN criteria CT scan	Higher IL-1β in VaD, AD, and CDND vs CON (*p* < 0.005) Higher TNF-α in VaD and AD vs CON (*p* < 0.05), and in VaD vs AD (*p* < 0.03) Higher IL-6 in VaD vs AD (p < 0.03)
Tarkowski, 1999 (Sweden)	Patients with AD, VaD or cognitively healthy (CON)	AD: 34 VaD: 33 CON: 55 (25 with CSF assessment)	66.7 y	TNF-α, IL-1β, IL-6 [serum and CSF]	-	AD: according definition of “pure” AD by Wallin et al. (1994) VaD: NINDS- AIREN criteria CT scan	Higher TNF-α in the CSF of AD (*p* = 0.006) and VaD patients (*p* = 0.001) vs CON No difference in TNF-α serum level between AD, VaD and CON Higher TNF-α in CSF than in the sera in patients with AD (*p* = 0.039) and with VaD (*p* = 0.007) Higher serum IL-6 in VaD vs CON (*p* < 0.0001) and vs CSF IL-6 (*p* = 0.002) No difference in CSF and serum IL-6 and IL-1β levels between AD and VaD
Wehr, 2019 (Poland)	Patients with different types of dementia, or cognitively healthy (CON)	AD: 166 VaD: 85 MIX: 149 CON: 180	73.3 y	IL-6, hs-CRP, chitotriosidase activity, paraoxonase-1 [serum]	-	AD: NINCDS-ADRDA criteria VaD: NINDS- AIREN criteria CT or MRI scan	Higher IL-6 in the whole dementia group and in the VaD, MIX groups vs CON Higher hs-CRP in the VaD group Higher chitotriosidase activity in VaD and MIX but not in AD
Vishnu, 2017 (India)	Patients with different types of dementia or cognitive impairment	AD: 41 VaD: 11 MCI: 11 Mild VCI: 5	-	Fibrinogen, D-dimer, IL-6, CRP [plasma]	-	AD: Dubois criteria, VaD: DSM IV criteria MCI/mild VCI: NIA-AA criteria MRI, FDG-PET and CSF biomarkers	No difference in IL-6 and CRP between AD and VaD Higher fibrinogen in VaD Higher D-dimer levels in VaD
Mancinella, 2009 (Italy)	Patients with different types of dementia, or cognitively healthy (CON)	AD: 35 VaD: 64 CON: 99	83.5 y	Fibrinogen, hs-CRP [plasma]	-	AD: NINCDS-ADRDA criteria VaD: NINDS- AIREN criteria CT or MRI scan	Higher hs-CRP in dementia group vs control and in AD vs VaD

Abbreviations: *AD* Alzheimer’s disease, *ADDTC* Alzheimer’s Disease Diagnostic and Treatment Centers, *ADL* activities of daily living scale, *CDR* clinical dementia rating, *CON* controls, *CRP* c-reactive protein, *CSF* cerebrospinal fluid, *CT* computed tomography, *CVND* cerebrovascular disease but without dementia, *FDG-PET* 18fluoro-2-deoxyglucose positron emission tomography, *IL* interleukin, *HIS* Hachinski Ischemic Scale, *hs-CRP* high sensitive c-reactive protein, *MCI* mild cognitive impairment, *MIX* mixed dementia, *MoCA* montreal cognitive assessment, *MRI* magnetic resonance imaging, *NIA-AA* National Institute on Aging-Alzheimer’s Association, *NINCDS-ADRDA* National Institute of Neurological and Communicative Disorders and Stroke-Alzheimer’s Disease and related Disorders Association, *NINDS-AIREN* National Institute of Neurological Disorders and Stroke–Association Internationale pour la Recherche et l’Enseignement en Neurosciences, *SCI* subjective cognitive impairment, *SPECT* single photon emission computed tomography, *sTNF-RI* soluble tumor necrosis factor receptor type I, *TNF* tumor necrosis factor, *VaD* vascular dementia, *VCI* vascular cognitive impairment

**Table 2:** Characteristics of longitudinal studies investigating relationship between inflammatory markers and vascular dementia

Study (country)	Mean follow-up	Population	Sample size	Mean age at baseline	Inflammatory biomarkers	Covariates	Diagnostic criteria	Main results
Miwa, 2016 (Japan)	7.5 y	Participants form Osaka Follow-up Study for Carotid Atherosclerosis, Part 2 (OSACA2) on high CV risk subjects in primary and secondary prevention with MMSE score ≥ 24 and 0 on the CDR	803	67.0 y	IL-6, CRP [serum]	Age, sex, APO-Eε4, education, variants of IL-6 receptor gene (rs2228145)	AD: NINCDS-ADRDA criteria VaD: NINDS- AIREN criteria MRI scan	Higher IL-6 significantly associated with VaD (RR: 1.95, 95% CI: 1.15–3.19, *p* = 0.017), but this association disappeared when further adjusting for IL-6 receptor gene variant rs2228145
Gallacher, 2010 (England)	17.3 y	Men free of vascular disease and not taking anticoagulants at baseline	865	From 45 to 59 y	hs-CRP, IL-6, fibrinogen, plasma viscosity, WBC, α2-macroglobulin, α1-antitrypsin [plasma]	Age, social class, systolic pressure, BMI, smoking status, total cholesterol, and alcohol consumption	AD: NINCDS-ADRDA criteria VaD: NINDS-AIREN criteria Rosen-modified HIS	Higher fibrinogen associated with VaD (HR: 1.68, 95% CI: 1.02–2.76) Other inflammatory markers not associated with VaD
Ravaglia, 2007 (Italy)	3.7 y	Participants aged 65 years and older free of dementia from the Conselice Study of Brain Ageing	804	73.6 y	hs-CRP, IL-6 [serum] α1-antichymotrypsin [plasma]	Age, gender, education, APO-E genotype, history of stroke and cardiovascular disease, physical activity, and BMI	AD: NINCDS-ADRDA criteria VaD: NINDS-AIREN criteria CT scan	Higher CRP associated with VaD (HR: 2.93, 95% CI: 1.39–6.18) Combination of high CRP and high IL6 associated with risk of VaD (HR: 2.56, 95% CI: 1.21–5.50)
Engelhart, 2004 (Netherlands)	6–9 y	Participants aged 55 years and older free of dementia from Rotterdam Study	727	71.7 y	ACT, CRP, IL-6, sICAM-1, and sVCAM-1 [plasma]	Age, gender, education, smoking, BMI, diabetes mellitus, use of anti-inflammatory medication, and atherosclerosis	AD: NINCDS-ADRDA criteria VaD: NINDS-AIREN criteria	ACT (RR: 2.48) and CRP (RR: 1.31) associated with increased risk of Va Levels of IL-6, sICAM-1 and sVCAM-1 not associated with VaD
Schmidt, 2002 (United States)	25 y	Nested case–control study from Honolulu-Asia Aging Study in Japanese American men	1,050	29.2 y	hs-CRP [serum]	Education, smoking, midlife average cholesterol, midlife blood pressure, age, years of follow-up, APO-Eε4, BMI, stroke, coronary heart disease, left ventricular hypertrophy, atrial fibrillation, diabetes mellitus, ABI at the time of dementia assessment	AD: NINCDS-ADRDA criteria VaD: California Alzheimer’s Disease Diagnostic and Treatment Centers criteria	Compared with lowest hs-CRP quartile (< 0.34 mg/L), men in the upper three quartiles had a threefold higher risk for all dementias, AD, and VaD. For VaD, the risk increased with increasing quartile
Hsu, 2017 (Taiwan)	11 y	Participants aged 65 years and older free of dementia from the Elderly Nutrition and Health Survey in Taiwan	1,436	73.2 y	CRP [serum]	Age, gender, education, BMI, use of sleep medication, alcohol consumption, and diastolic blood pressure	Not specified (review of ICD-9-CM code from medical records)	Higher CRP associated with higher risk of VaD (HR: 2.09; 95% CI: 1.01–4.32) but not AD
Van Oijen, 2005 (Netherlands)	5.7 y	Participants aged 55 years and older free of dementia from Rotterdam Study	6,713	69.5 y	Fibrinogen, hs-CRP [plasma]	Cardiovascular risk factors, presence of apoEε4, previous stroke, white blood cell count, and fibrinogen	AD: NINCDS-ADRDA criteria VaD: NINDS-AIREN criteria	High fibrinogen levels associated with higher risk of both AD (HR: 1.25, 95% CI: 1.04–1.49) and VaD (HR: 1.76, 95% CI: 1.34–2.30) Higher levels of CRP not associated with higher risk of dementia

Abbreviations: *ABI* ankle-brachial index, *ACT* α1-antichymotrypsin, *AD* Alzheimer’s disease, *APOE* apolipoprotein E, *BMI* body mass index, *CI* confidence interval, *CRP* c-reactive protein, *CT* computed tomography, *IL* interleukin, *HIS* Hachinski Ischemic Scale, *HR* hazard ratio, *hs-CRP* high sensitive c-reactive protein, *MRI* magnetic resonance imaging, *NINCDS-ADRDA* National Institute of Neurological and Communicative Disorders and Stroke-Alzheimer’s Disease and related Disorders Association, *NINDS-AIREN* National Institute of Neurological Disorders and Stroke–Association Internationale pour la Recherche et l’Enseignement en Neurosciences, *RR* relative risk, *sICAM-1* soluble forms of intercellular adhesion molecule-1, *sVCAM-1* vascular cell adhesion molecule-1, *VaD* vascular dementia

### Quality assessment

We evaluated distribution of the risk of bias across the 20 studies included in the quantitative synthesis. The quality of the included studies ranged from medium to high, with comparability for case–control study and ascertainment of outcome for cohort studies as the major concerns for potential sources of bias (Supplementary Table 1).

### Blood inflammatory markers

#### Interleukin-6 and vascular dementia

**Fig. 2:**
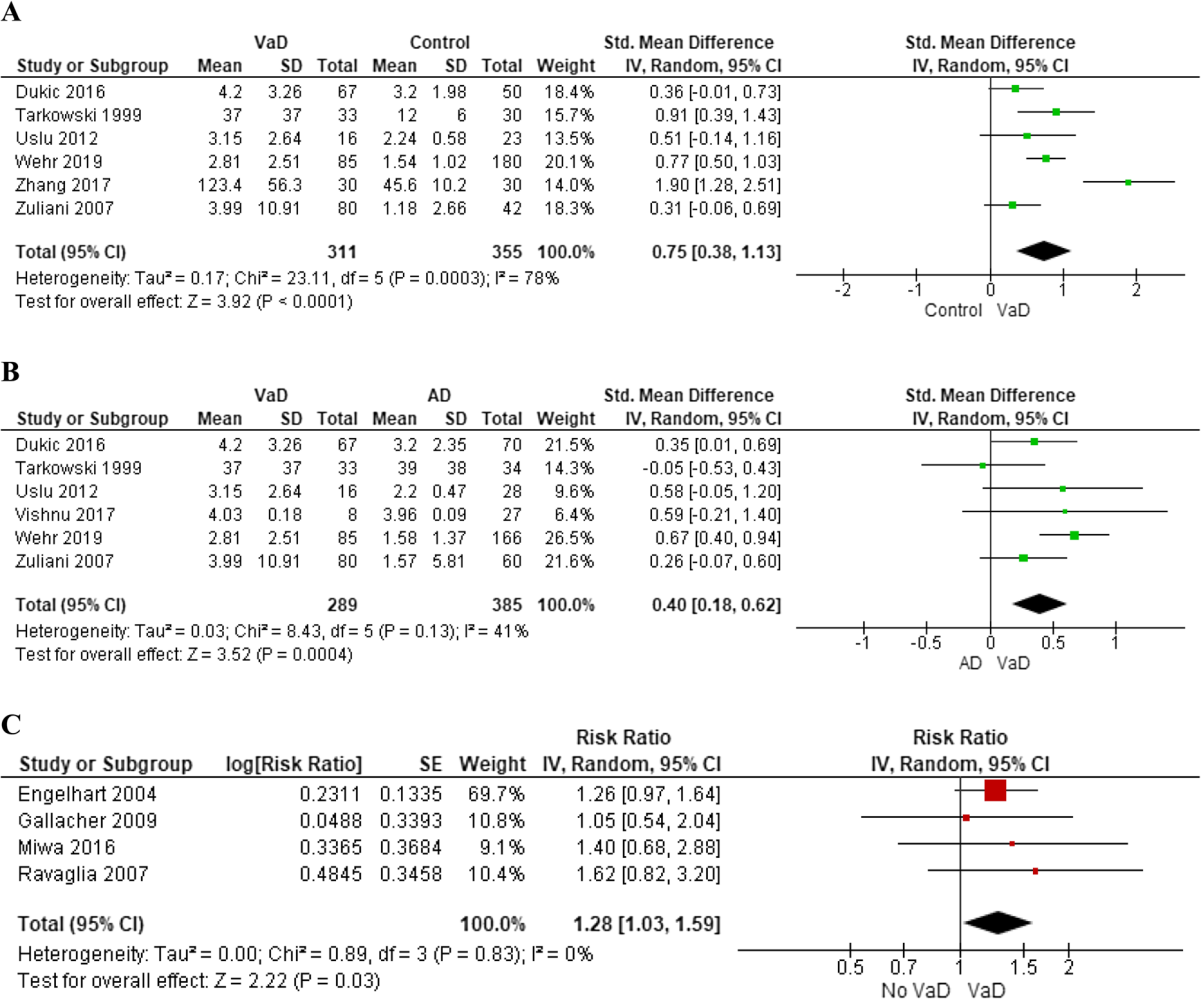
Difference in blood interleukin-6 levels between subjects with vascular dementia and controls (**A**) or those with Alzheimer’s disease (**B**); pooled hazard ratios for interleukin-6 and incident vascular dementia (**C**)

Six studies investigated serum or plasma IL-6 levels in 311 subjects with VaD compared to 355 healthy controls [19–21, 27–29]. IL-6 levels were significantly higher in people with VaD compared to healthy subjects (SMD: 0.75, 95% CI: 0.38 to 1.13) with evidence of high heterogeneity across the studies (*I*^2^ = 78%, *p* < 0.001) (Fig. 2A). No small study effect was detected at Egger’s test (*p* = 0.475) (Supplementary Fig. 1). In a sensitivity analysis, we excluded the study by Zhang and colleagues [19], which determined asymmetry at funnel plot. The levels of IL-6 remained still significantly higher in VaD patients compared to controls (SMD: 0.57, 95% CI: 0.33 to 0.81), but the heterogeneity was reduced (*I*^2^ = 42%, *p* = 0.14). Results were confirmed also removing the studies with low-quality assessment at NOS [19, 20, 28] (SMD: 0.56, 95% CI: 0.24 to 0.88), with low heterogeneity (*I*^2^ = 48%, *p* = 0.14).

Six studies investigated difference in serum or plasma IL-6 levels between 289 subjects with VaD and 385 subjects affected by AD [20, 21, 27–30]. IL-6 levels were significantly higher in subjects with VaD compared to those with AD (SMD: 0.40, 95% CI: 0.18 to 0.62), with low heterogeneity (*I*^2^ = 41%, *p* = 0.13) (Fig. 2B) and no evidence of small study effect at Egger’s test (*p* = 0.662) (Supplementary Fig. 2). Findings were confirmed by a sensitivity analysis including only high-quality studies (NOS ≥ 7) [21, 27, 29] (SMD: 0.50, 95% CI: 0.22 to 0.78, *I*^2^ = 41%, *p* = 0.18).

Four studies explored the risk of incident VaD among 3,345 cognitive healthy subjects over a mean follow-up of 8.6 years (range: 4–17 years) [32–35]. Out of the four studies, one was nested case–control [35], and the other three were cohort studies [32–34]. Median IL-6 levels at baseline ranged from 1.17 to 2.20 pg/ml [33]. For one-unit increase in ln IL-6 levels, the rate of VaD rose by 28% (RR: 1.28, 95% CI: 1.03 to 1.59) with no evidence of heterogeneity (*I*^2^ = 0%, *p* = 0.83) (Fig. 2C) or small study effect (*p* = 0.739) across the studies (Supplementary Fig. 3). Results were consistent after removing one study with low-quality assessment [33] (RR: 1.31, 95% CI: 1.04 to 1.65, *I*^2^ = 0%, *p* = 0.78).

#### C-reactive protein and vascular dementia

**Fig. 3:**
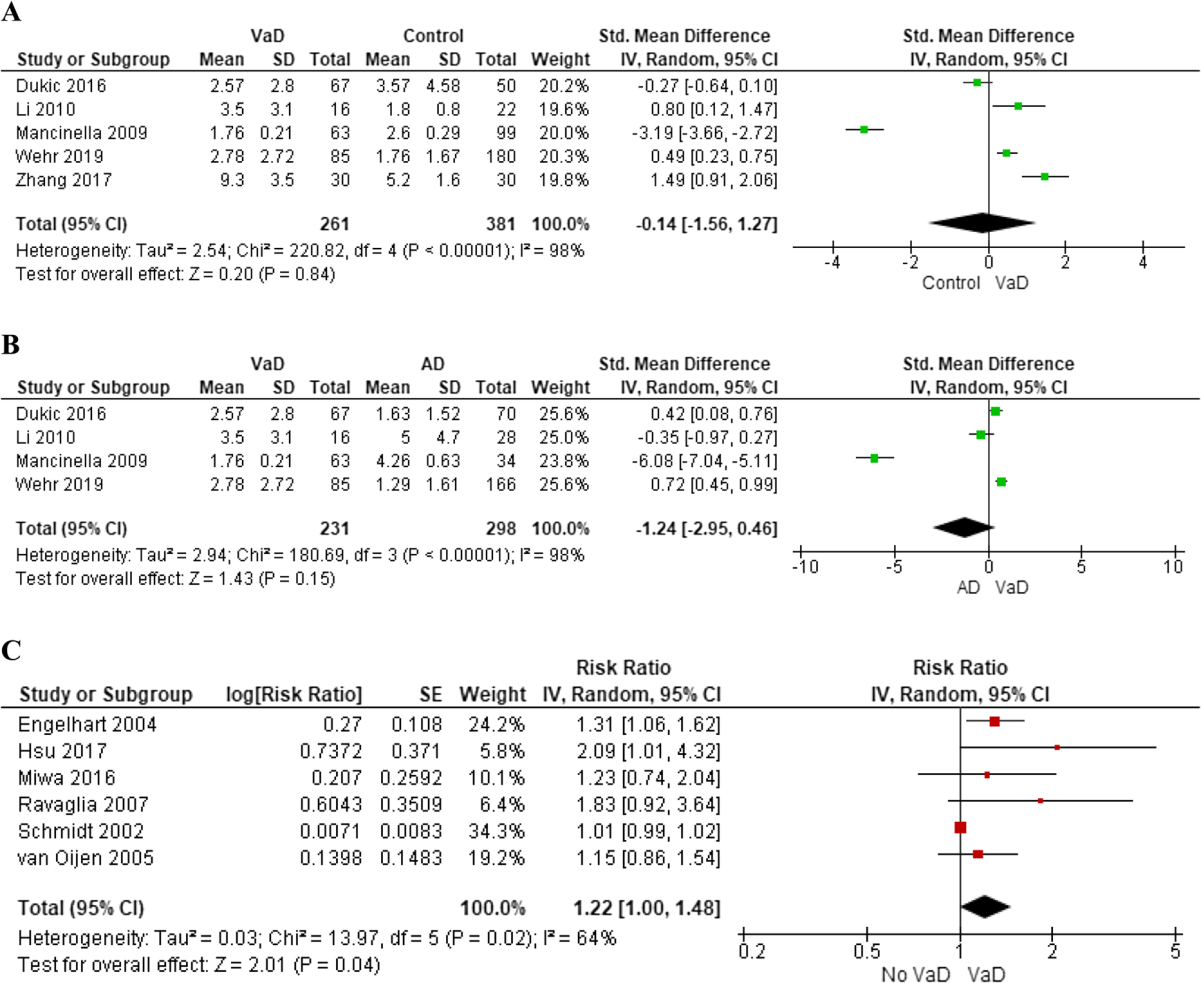
Difference in blood C-reactive protein levels between subjects with vascular dementia and controls (**A**) or those with Alzheimer’s disease (**B**); pooled hazard ratios for C-reactive protein and incident vascular dementia (**C**)

Five studies analyzed differences in CRP levels between a total of 261 subjects with VaD and 381 healthy controls [19, 20, 22, 29, 31]. CRP levels did not significantly differ compared to healthy controls (SMD: − 0.14, 95% CI: − 1.56 to 1.27) with high heterogeneity across the study (*I*^2^ = 98%, *p* < 0.001) (Fig. 3A), but no small study effect (*p* = 0.829) (Supplementary Fig. 4). Removing the study by Mancinella and colleagues [31] that, at visual inspection of funnel plot, led to marked asymmetry, levels of CRP in VaD compared to controls were still not significantly different (SMD: 0.59, 95% CI: − 0.06 to 1.25), with a modest reduction of heterogeneity (*I*^2^ = 89%, *p* < 0.001). We also performed sensitivity analysis excluding low-quality studies [19, 20], but the inconsistency across the studies was not reduced (*I*^2^ = 99%, *p* < 0.001), suggesting presence of other potential sources of heterogeneity (e.g., CRP assessment method).

Four studies compared CRP levels between 231 patients with VaD and 298 AD patients [20, 22, 29, 31]. No significant difference was found (SMD: − 1.24, 95% CI: − 2.95 to 0.46), with evidence of high heterogeneity across the studies (*I*^2^ = 98%, *p* < 0.001) (Fig. 3B). No small study effect was detected at Egger’s test (*p* = 0.096) (Supplementary Fig. 5). In a sensitivity analysis, we removed the study by Mancinella and colleagues [31], which determined remarkable asymmetry at funnel plot, but results did not change (SMD: 0.34, 95% CI: − 0.16 to 0.83, *I*^2^ = 80%, *p* = 0.006). Also removing the study with poorer quality assessment [20] did not reduced the very high heterogeneity (*I*^2^ = 99%, *p* < 0.001).

Six high-quality studies with an overall population of 11,679 cognitive healthy subjects explored the risk of incident VaD during a mean follow-up of 10 years (range: 4–25 years) [32, 34–38]. Two were nested case–control studies [35, 36] and four were cohort studies [32, 34, 37, 38]. All but two studies [32, 37] measured high-sensitivity CRP; three studies used immunonephelometry [34, 35, 38], and the remaining studies used ELISA methodology [36] or immunoturbidimetric assay [32, 37]. Median CRP levels at baseline ranged from 0.57 [36] to 17.2 mg/l [35]. No significant increase of VaD risk was observed for one-unit change in ln CRP (RR: 1.22, 95% CI: 1.00 to 1.48) with significant heterogeneity between the studies (*I*^2^ = 64%, *p* = 0.02) (Fig. 3C), and evidence of small study effect at Egger’s test (*p* = 0.009) (Supplementary Fig. 6).

#### Tumor necrosis factor-α and vascular dementia

**Fig. 4:**
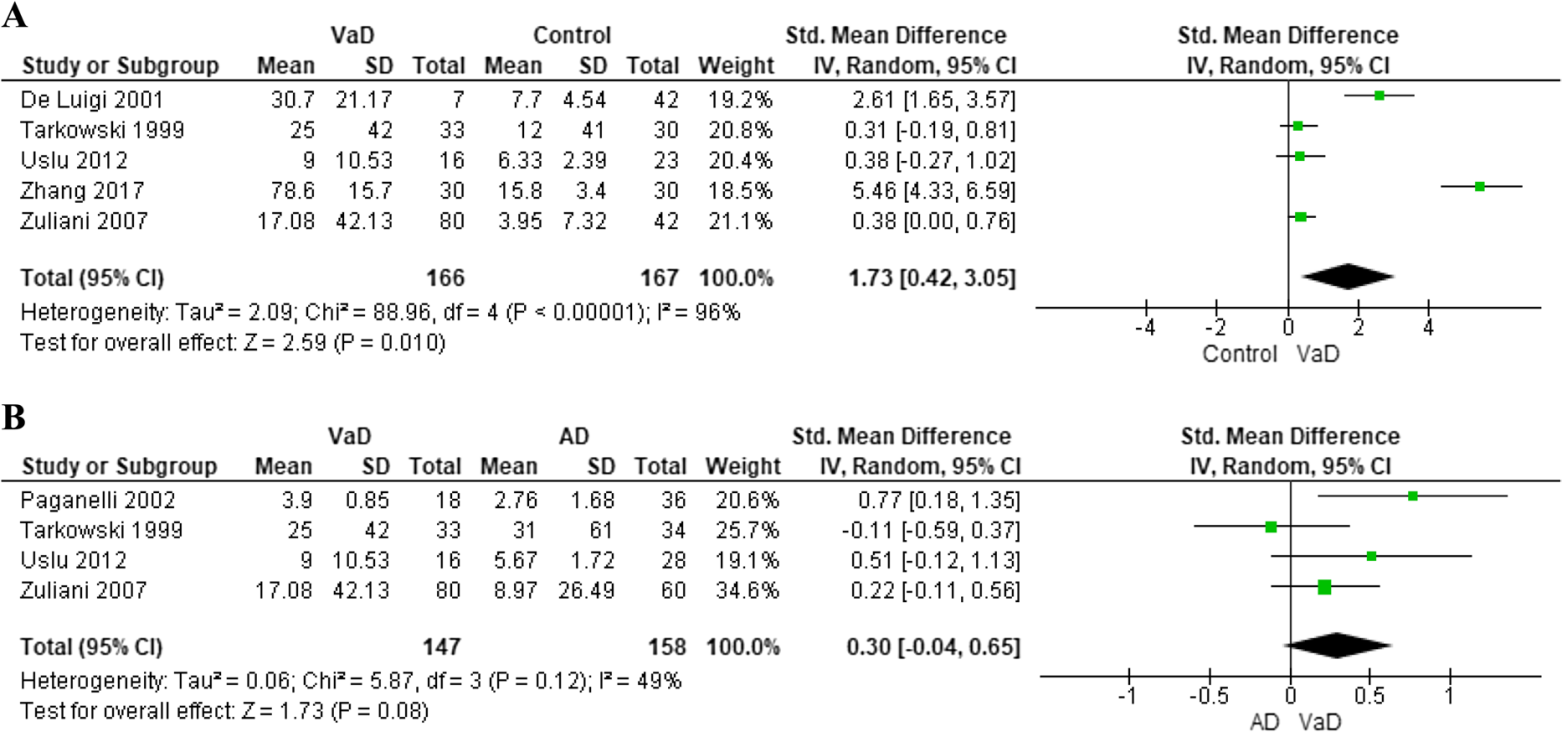
Difference in blood tumor necrosis factor-α levels between subjects with vascular dementia and controls (**A**) or those with Alzheimer’s disease (**B**)

Five studies analyzed differences in tumor necrosis factor (TNF)-α levels between a total of 166 subjects with VaD and 167 healthy controls [19, 21, 26–28]. TNF-α levels were more elevated in VaD patients compared to healthy controls (SMD: 1.73, 95% CI: 0.42 to 3.05) with evidence of high heterogeneity across the studies (*I*^2^ = 96%, *p* < 0.001) (Fig. 4A), and no small study effect (*p* = 0.06) (Supplementary Fig. 7). Excluding the studies by Zhang and colleagues [19] and De Luigi and colleagues [26] that, at visual inspection of funnel plot, led to marked asymmetry, the higher levels of TNF-α in VaD compared to controls were still confirmed (SMD: 0.36, 95% CI: 0.09 to 0.63), but the heterogeneity was reduced (*I*^2^ = 0%, *p* = 0.97). These findings were further confirmed performing sensitivity analysis with the exclusion of all low-quality studies [19, 26, 28] (SMD: 0.38, 95% CI: 0.05 to 0.70, *I*^2^ = 0%, *p* = 0.99).

Moreover, four studies compared circulating concentration of TNF-α between 147 VaD and 158 AD patients [21, 25, 27, 28]. No significant difference was detected among these two subgroups (SMD: 0.30, 95% CI: − 0.04 to 0.65), with low heterogeneity across the studies (*I*^2^ = 49%, *p* = 0.12) (Fig. 4B) and no evidence of small study effect (*p* = 0.507) (Supplementary Fig. 8). Removing low-quality studies [25, 28], the results were confirmed, also reducing the heterogeneity (SMD: 0.30, 95% CI: − 0.04 to 0.65, *I*^2^ = 0%, *p* = 0.43). No study analyzed correlation between blood TNF-α levels and incident VaD.

#### Interleukin-6 in cerebrospinal fluid and vascular dementia

**Fig. 5:**
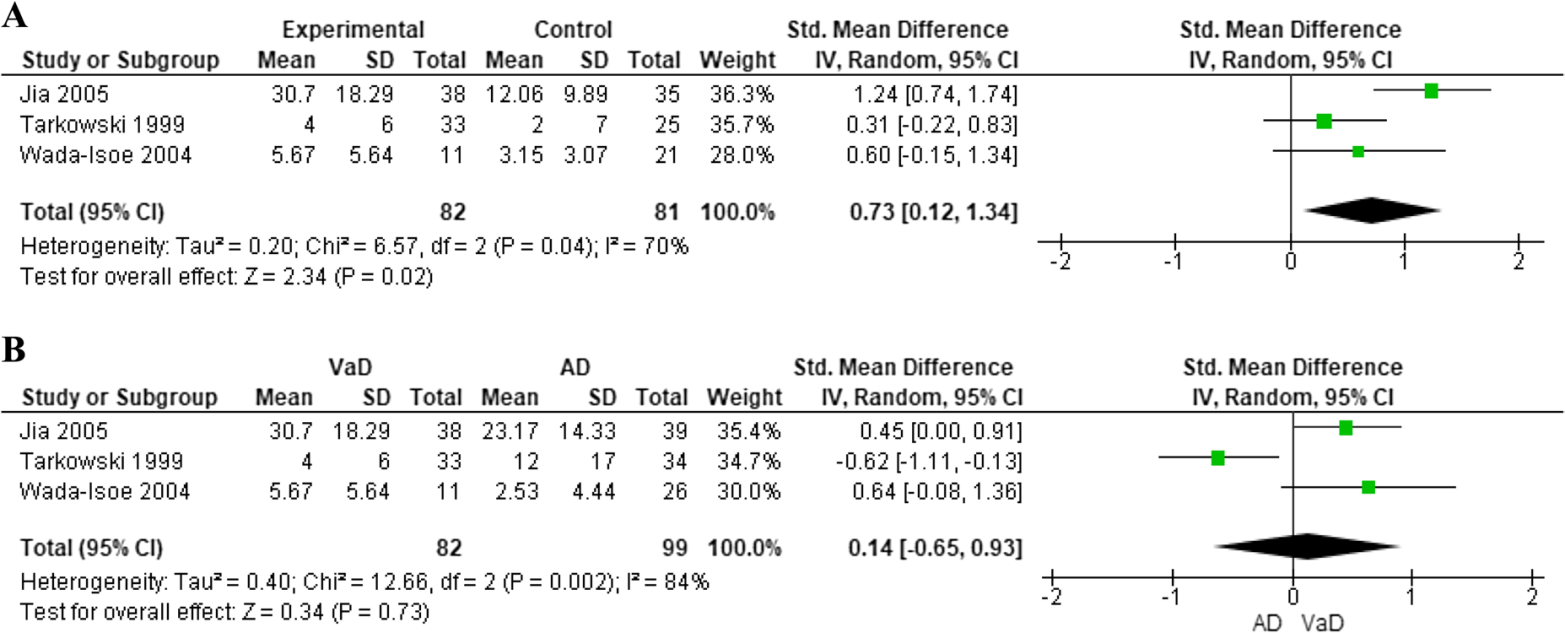
Difference in cerebrospinal fluid interleukin-6 levels between subjects with vascular dementia and controls (**A**) or those with Alzheimer’s disease (**B**)

For quantitative synthesis, only three studies were eligible which compared IL-6 levels in the CSF between 82 patients with VaD, 99 with AD, and 81 healthy subjects [23, 24, 28]. IL-6 was significantly higher in people with VaD compared to healthy subjects (SMD: 0.73, 95% CI: 0.12 to 1.34) (Fig. 5A), but not compared to AD patients (SMD: 0.14, 95% CI: − 0.65 to 0.93) (Fig. 5B). Despite no evidence of small study effect (*p* = 0.830 for VaD vs healthy controls, *p* = 0.800 for VaD vs AD) (Supplementary Figs. 9 and 10), high heterogeneity (*I*^2^ = 70%, *p* = 0.04 for VaD vs healthy controls, *I*^2^ = 84%, *p* = 0.002 for VaD vs AD) (Fig. 5A and B, respectively), together with poor overall quality of the studies, limited the reliability of these findings.

## Discussion

In the present systematic review and meta-analysis, we investigated the usefulness of blood and CSF inflammatory biomarkers for VaD diagnosis. We found that, compared to healthy subjects, a moderate to large elevation of both blood IL-6 and TNF-α levels was associated with VaD diagnosis. However, only blood IL-6 concentrations significantly differed between VaD and AD subjects such that patients with VaD had small to moderate elevation of IL-6 compared to those with AD. Moreover, we found that each unit increase of IL-6 levels predicted 28% higher risk of VaD. In the CSF of VaD patients, IL-6 levels were significantly higher than in healthy subjects, but no difference was detected compared to AD patients. Data from CSF should be taken with caution due to high inconsistency related to the still limited number of studies with relatively small sample size.

Present findings might suggest that among inflammatory markers, circulating IL-6 levels could be a useful biomarker able to differentiate across healthy, VaD, and AD subjects. A recent meta-analysis by Ng and colleagues showed that blood inflammatory markers, including IL-6, were not significantly different between AD patients and controls [39]. However, evidence from both cross-sectional and prospective studies highlight that higher IL-6 levels are related with poorer cognitive performance [40, 41] and faster cognitive decline [42, 43]. The relationship between cognitive impairment and inflammation in VCID is partly explained by the existence of a clear association between inflammatory status, atherosclerosis, and prothrombotic conditions [44]. Compared to previous meta-analytic findings that did not evidence any significant association between circulating CRP and IL-6 levels and future risk of AD [12, 13], we found a positive linear relationship between blood IL-6 and risk of incident VaD.

In the present meta-analysis, among CSF inflammatory biomarkers, only IL-6 had enough studies to be included in quantitative synthesis. However, other inflammatory markers in CSF are under investigation. For example, few reports showed that TNF-α levels in the CSF were higher than in sera among subjects with dementia, suggesting an intrathecal synthesis of this cytokine [28]. Assessment of the soluble forms of TNF-α receptors (sTNFR1 and 2) which may provide more accurate information about activation of the TNF-α system revealed that patients with mild cognitive impairment (MCI) who converted to VaD had higher concentrations of these biomarkers compared to those who converted in AD [45]. Biomarkers of microglial activation in CSF, which are related to neuroinflammation (i.e., YKL-40 and calcium binding protein B), were not able to differentiate between AD and VaD patients [46]. However, Olsson and colleagues showed that in subjects with MCI followed over 5.7 years, higher levels of YKL-40 and sCD14 in CSF predicted conversion to VaD but not to AD [47]. Further well-conducted studies are warranted to draw conclusion on reliability of inflammatory markers from CSF in VaD diagnosis.

Our findings might suggest that systemic inflammation contributes to VCID. Studies on brain biopsies showed controversial results on the contribution of inflammatory mechanisms in the pathogenesis of VaD [48–50]. It has been hypothesized that different types of cerebral small vessel disease (SVD) might be mechanistically linked to different forms of inflammation [51]. Cerebral SVD represents one of the most common neuropathological features of VCID [52]. It has been shown that biomarkers of systemic inflammation, like IL-6, may be associated with a specific form of SVD, the cerebral amyloid angiopathy (CAA) also known as type 2 SVD, which involves lobar regions and the centrum semiovale [51]. Conversely, sustained elevation over time of biomarkers of systemic inflammation is longitudinally associated with SVD progression [51].

Among different inflammatory biomarkers, we found a preeminent role of IL-6 in the diagnosis of VaD. Preclinical and clinical studies have demonstrated that during aging, in endothelial and smooth muscle cells, there occurs an overexpression of genes coding for inflammatory cytokines, chemokines, adhesion molecules, and other proinflammatory mediators, leading to the development of a proinflammatory microenvironment that promotes vascular dysfunction [53, 54]. Moreover, higher inflammatory markers may underlie a damage of neurovascular unit [55]. Indeed, inflammatory and oxidative injuries may alter neurons and white matter function by interfering with neurovascular coupling [56]. This process exacerbates tissue hypoxia, by contrasting proliferation, migration, and differentiation of oligodendrocyte stem cells and by compromising mechanisms of reparation of damage in the white matter [57]. In addition, the activation of leukocytes and the release of inflammatory cytokines and cell adhesion molecules, which have been observed in patients with hypertension, may induce a dysregulation of the signaling of angiotensin II [58]. This dysregulation may lead to the impairment of the modulation of cerebral perfusion in response to blood pressure variations [59]. Specifically, IL-6 is involved in atherosclerosis through a large variety of pathways leading to plaque formation, from the stimulation of the acute-phase reactants and coagulation factors synthesis in the liver to the promotion of proliferation and differentiation of leukocytes and the activation of endothelial cells [60]. The latter respond to the IL-6 stimuli by releasing chemokines and increasing the expression of cellular adhesion molecules as the intercellular adhesion molecule 1 (ICAM-1), which is involved in the adhesion and transmigration of circulating leukocytes [61]. Promising perspectives come from other blood proinflammatory biomarkers as midregional proenkephalin A (MR-PENK A), mainly associated with pain sensation, cardiac function, and immunity, which has been positively associated with increased risk of VaD [62].

Despite data from randomized controlled trials are still scarce, targeting proinflammatory pathways may be a promising approach for the prevention of cardiovascular diseases and potentially VCID. Among eligible pharmacological strategies geared toward systemic inflammation, the inhibition of TNF-α signaling or the treatment with the IL-6 inhibitor tocilizumab determined an improvement of endothelial function assessed by means of flow-mediated dilatation [63, 64]. Also findings from COLCOT trial demonstrated the effectiveness of the colchicine in secondary cardiovascular prevention after myocardial infarction [65]. On the other hand, the administration of low-dose methotrexate did not result in fewer cardiovascular events compared to placebo [66]. Great interest has been aroused by the effect of a therapeutic monoclonal antibody targeting IL-1β, canakinumab, whose administration led, in a large cohort of patients with previous myocardial infarction, to a significantly lower rate of recurrent cardiovascular events [67]. Nevertheless, canakinumab was not approved for cardiovascular disease prevention, due to increased risk of fatal infections. Also, the statins, beyond their lipid-lowering effect, have well-characterized anti-inflammatory properties including the inhibition of the formation of isoprenoids and proinflammatory mediators, and the subsequent reduction of asymmetrical dimethylarginine, implicated in endothelial dysfunction [68, 69]. Noteworthy, several randomized controlled trials demonstrated that patients taking statins had a significant reduction of CRP levels [70, 71], but evidence of a protective role of statins against VCID are still insufficient [72].

A few preclinical studies explored the effectiveness of other compounds with anti-inflammatory properties for VCID prevention. The angiotensin-(1–7) glycosylated mas receptor agonist demonstrated the ability to restore visual-spatial memory in a murine model of VCID [73]. Another molecule, the N-palmitoylethanolamide-oxazoline, reduced in mice the histological alterations typical of VCID and improved behavioral disorders through neuroprotective and anti-inflammatory activity [74]. Furthermore, treatment with resveratrol which has well-known anti-inflammatory and antioxidant properties was associated, in a rodent model of VaD, to better vascular reactivity and reduction of cognitive decline [75]. Future preclinical and clinical studies should test if strategies targeting chronic inflammation and, in particular, blood IL-6 could have a role in reducing incidence of VCID or slow down its progression.

To the best of our knowledge, this is the first meta-analysis exploring the reliability of few well-accepted inflammatory biomarkers (IL-6, CRP, and TNF-α) for differential diagnosis between VaD and AD. This distinguishes our findings from those of other previous systematic reviews and meta-analyses that assessed only the association between inflammation and overall dementia or AD [12, 13]. The present study has also some limitations. First, despite the strict inclusion/exclusion criteria, there is wide heterogeneity observed across the studies, due to potential several reasons: (a) different measurement platforms, (b) small sample size per each study, (c) different case adjudication methods, (d) presence of subjects in different VCID stages in cross-sectional studies, or (e) different lengths of follow-up in longitudinal studies. Second, several studies had relatively small sample sizes that could potentially lead to overestimation of effects. Nevertheless, we performed sensitivity analysis excluding the studies at higher risk of publication bias, and we did not detect any significant change in the results. Third, for most of the studies, the assessment of inflammatory state was based only on a single value of the biomarker which could lead to a misclassification of exposure. Fourth, although we included only studies in which VaD and AD diagnosis were based on internationally validated criteria, misclassification of outcome should be accounted given the different methods used for diagnosis and the few studies including a confirmation of diagnosis by imaging techniques. In this regard, it is worthy of mention that whenever specified, subjects with mixed dementia were excluded. Fifth, the included studies adjusted the analysis for different factors; therefore, there could be unmeasured confounders associated with inflammation and dementia. Sixth, results on CSF biomarkers should be considered with caution due to limited number of included studies. Finally, the assays for biochemical measurements of serum or plasma IL-6, CRP, and TNF-α varied across the studies.

In conclusion, blood IL-6 levels might represent a useful biomarker of VCID, able to differentiate people with VaD from those with AD and to predict future VaD risk in healthy subjects. Further prospective, high-quality studies are warranted to test opportune IL-6 cutoffs for VCID diagnosis alone or in combination with other inflammatory biomarkers, the association of IL-6 levels with different stages across the VCID spectrum, and finally the usefulness in better characterization of mixed dementia. Ultimately, present findings should encourage promotion of preventive strategies targeting systemic inflammation in subjects with high cardiovascular risk.

## Supplementary Information

Below is the link to the electronic supplementary material.

Supplementary file1 (DOCX 85.8 KB)
