# Supplementary material for: Role of inflammatory markers in the diagnosis of vascular contributions to cognitive impairment and dementia: a systematic review and meta-analysis
Source: GeroScience. 2022 Apr 29;44(3):1373–92. doi: 10.1007/s11357-022-00556-w (PMC9213626; doi:10.1007/s11357-022-00556-w)
Supplement: Supplementary file 1 — Supplementary file1 (DOCX 85.8 KB) [file 11357_2022_556_MOESM1_ESM.docx]

Supplementary Figure 1. Funnel plot with pseudo 95% confidence intervals for studies

exploring differences in blood interleukin-6 levels between subjects with vascular dementia and controls


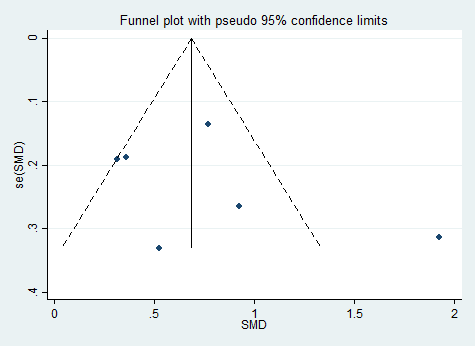


Supplementary Figure 2. Funnel plot with pseudo 95% confidence intervals for studies exploring differences in blood interleukin-6 levels between subjects with vascular dementia and Alzheimer’s disease


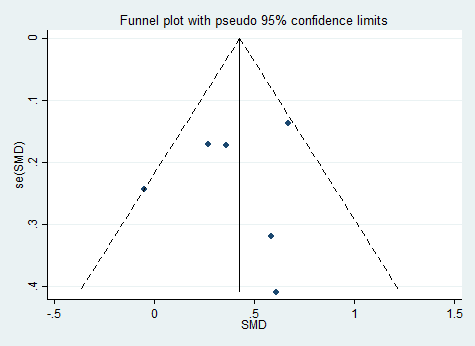


Supplementary Figure 3. Funnel plot with pseudo 95% confidence intervals for studies

exploring blood interleukin-6 levels and risk of incident vascular dementia


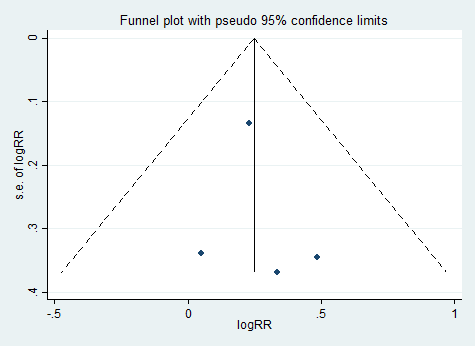


Supplementary Figure 4. Funnel plot with pseudo 95% confidence intervals for studies exploring differences in blood C-reactive protein levels between subjects with vascular dementia and controls


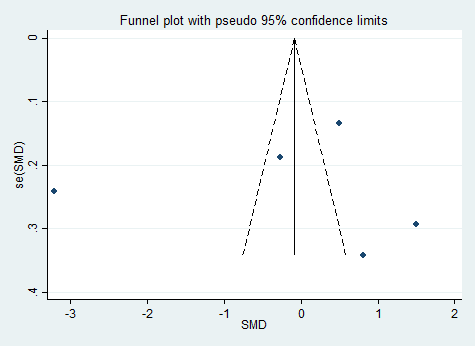


Supplementary Figure 5. Funnel plot with pseudo 95% confidence intervals for studies

exploring differences in blood C-reactive protein levels between subjects with vascular dementia and Alzheimer’s disease


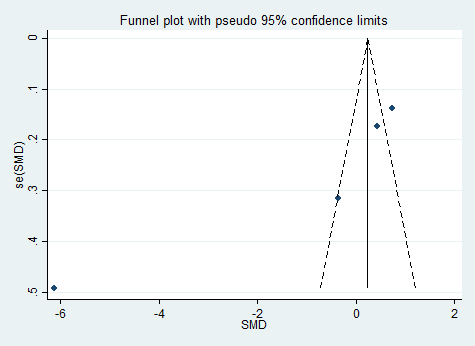


Supplementary Figure 6. Funnel plot with pseudo 95% confidence intervals for studies exploring blood C-reactive protein levels and risk of incident vascular dementia


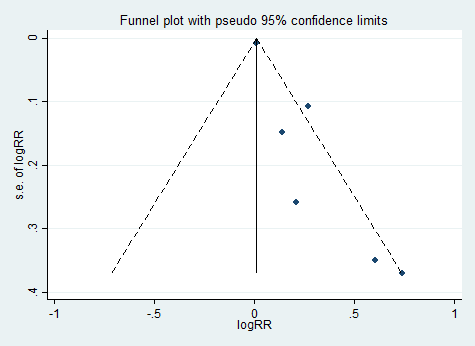


Supplementary Figure 7. Funnel plot with pseudo 95% confidence intervals for studies

exploring differences in blood tumor necrosis factor-α levels between subjects with vascular dementia and controls


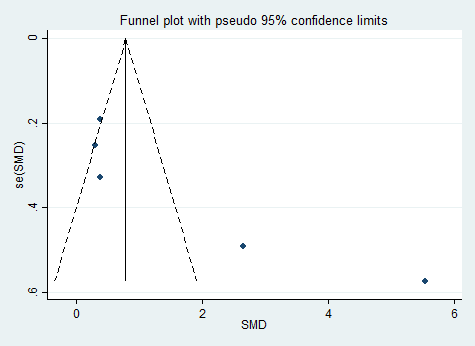


Supplementary Figure 8. Funnel plot with pseudo 95% confidence intervals for studies exploring differences in blood tumor necrosis factor-α levels between subjects with vascular dementia and Alzheimer’s disease


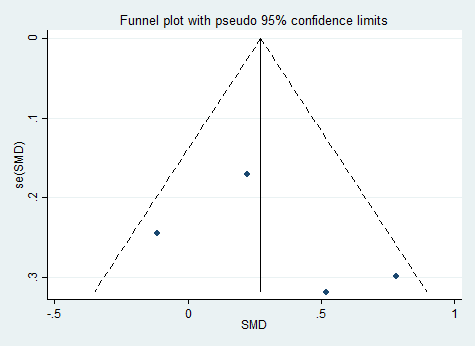


Supplementary Figure 9. Funnel plot with pseudo 95% confidence intervals for studies exploring differences in cerebrospinal fluid interleukin-6 levels between subjects with vascular dementia and controls


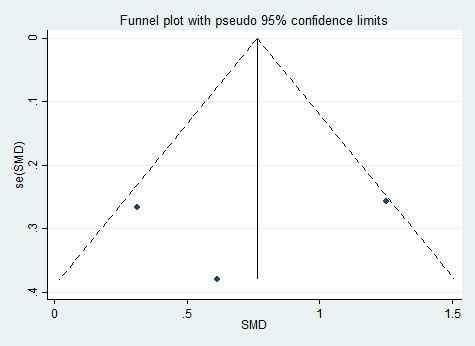


Supplementary Figure 10. Funnel plot with pseudo 95% confidence intervals for studies exploring differences in cerebrospinal fluid interleukin-6 levels between subjects with vascular dementia and Alzheimer’s disease


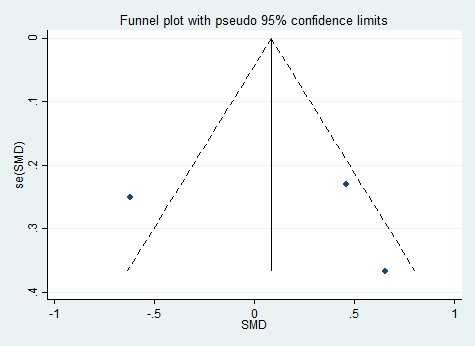


Supplementary Table 1. Newcastle-Ottawa quality assessment scale for evaluation of the quality of observational studies

|  |  | **Selection** | | | | **Comparability** | | **Outcome** | | |  |
| --- | --- | --- | --- | --- | --- | --- | --- | --- | --- | --- | --- |
| ID | First author, year | #1 | #2 | #3 | #4 | #5 | #6 | #7 | #8 | #9 | Total |
| **Case-control studies** | | | | | | | | | | | |
| 1 | De Luigi, 2001 |  | ★ |  | ★ | ★ |  |  | ★ | ★ | 5 |
| 2 | Dukic, 2016 | ★ | ★ |  | ★ |  |  |  | ★ | ★ | 5 |
| 3 | Jia, 2005 | ★ |  |  | ★ | ★ |  |  | ★ | ★ | 5 |
| 4 | Li, 2010 | ★ | ★ |  | ★ | ★ |  | ★ | ★ | ★ | 7 |
| 5 | Mancinella, 2009 | ★ | ★ | ★ | ★ | ★ | ★ | ★ | ★ | ★ | 9 |
| 6 | Paganelli, 2002 | ★ |  | ★ | ★ |  |  |  | ★ | ★ | 5 |
| 7 | Tarkowski, 1999 | ★ | ★ |  | ★ |  |  |  | ★ | ★ | 5 |
| 8 | Uslu, 2012 | ★ | ★ | ★ | ★ | ★ |  | ★ | ★ | ★ | 8 |
| 9 | Vishnu, 2017 | ★ |  | ★ | ★ |  |  | ★ | ★ |  | 5 |
| 10 | Wada-Isoe, 2004 | ★ |  |  | ★ |  |  |  | ★ | ★ | 4 |
| 11 | Wehr, 2019 | ★ | ★ | ★ | ★ |  |  | ★ | ★ | ★ | 7 |
| 12 | Zhang, 2017 | ★ | ★ | ★ | ★ |  |  |  | ★ | ★ | 6 |
| 13 | Zuliani, 2007 | ★ | ★ |  | ★ | ★ | ★ |  | ★ | ★ | 7 |
| **Cohort studies** | | | | | | | | | | | |
| 14 | Engelhart, 2004 | ★ | ★ | ★ | ★ | ★ | ★ | ★ | ★ | ★ | 9 |
| 15 | Gallacher, 2010 |  | ★ | ★ | ★ | ★ | ★ |  | ★ |  | 6 |
| 16 | Hsu, 2017 | ★ | ★ | ★ | ★ | ★ | ★ |  | ★ | ★ | 8 |
| 17 | Miwa, 2016 | ★ | ★ | ★ | ★ | ★ | ★ |  | ★ | ★ | 8 |
| 18 | Ravaglia, 2007 | ★ | ★ | ★ | ★ | ★ | ★ |  | ★ | ★ | 8 |
| 19 | Schmidt, 2002 | ★ |  | ★ | ★ | ★ | ★ | ★ | ★ | ★ | 8 |
| 20 | Van Oijen, 2005 | ★ | ★ | ★ | ★ | ★ | ★ | ★ | ★ | ★ | 9 |

#1. Adequate case definition/ Representativeness of patients with VaD

#2. Representativeness of the cases/ Selection of the Comparative patients without VaD #3. Selection of Controls/Ascertainment of VaD station/ distinction with AD

#4. Definition of Controls/ Demonstration that VaD was not present at start of study #5. Study controls for age

#6. Study controls for gender/other

#7. Ascertainment of exposure (case-control studies)/outcome (cohort studies)

#8. Same method of ascertainment for cases and controls/ Was follow-up long enough for VaD to occur

#9. Non-Response rate/Adequacy of follow up
